# Drug Repositioning: An Opportunity to Develop Novel Treatments for Alzheimer’s Disease

**DOI:** 10.3390/ph6101304

**Published:** 2013-10-11

**Authors:** Anne Corbett, Gareth Williams, Clive Ballard

**Affiliations:** Wolfson Centre for Age-Related Diseases, Guy’s Campus, King’s College London, London SE1 1UL, UK; E-Mails: anne.corbett@kcl.ac.uk (A.C.), gareth.2.williams@kcl.ac.uk (G.W.)

**Keywords:** Alzheimer’s, repositioning, treatment, drug

## Abstract

Alzheimer’s Disease (AD) is the most common cause of dementia, affecting approximately two thirds of the 35 million people worldwide with the condition. Despite this, effective treatments are lacking, and there are no drugs that elicit disease modifying effects to improve outcome. There is an urgent need to develop and evaluate more effective pharmacological treatments. Drug repositioning offers an exciting opportunity to repurpose existing licensed treatments for use in AD, with the benefit of providing a far more rapid route to the clinic than through novel drug discovery approaches. This review outlines the current most promising candidates for repositioning in AD, their supporting evidence and their progress through trials to date. Furthermore, it begins to explore the potential of new transcriptomic and microarray techniques to consider the future of drug repositioning as a viable approach to drug discovery.

## 1. Introduction

Dementia affects 35 million people worldwide, and this is set to rise to 115 million by 2050 [1]. This devastating condition incurs an enormous personal cost to those affected and a worldwide financial cost in 2010 estimated at $604 billion [1]. Alzheimer’s Disease (AD) is the most common cause of dementia, affecting up to two thirds of people affected. The progressive nature of the cognitive decline that occurs in people with AD lead to complex treatment and care needs which often require intensive care. Effective care and treatment is vital in ensuring people are able to live well with the condition. AD therefore represents a major and increasing public health concern and there is an urgent imperative to develop more effective therapies to treat and delay the onset of the disease.

AD is characterised by the accumulation of neuritic plaques consisting of the β-amyloid (Aβ) peptide and neurofibrillary tangles (NFT) comprised of hyperphosphorylated tau protein. This Alzheimer pathology is associated with disruption of synaptic and neuronal function leading to progressive loss of neurons and brain volume. The precise disease mechanism and pathways in AD remain unclear and there is some controversy regarding the role of specific toxic substrates [2]. The most prevailing amyloid cascade hypothesis’, which postulates the role of Aβ fragments as a toxic catalyst for plaque accumulation and subsequent development of NFT, has been called into question due to the lack of evidence to support it. It is also likely that the complexity of the disease pathway is heightened through the role of inflammation, mitochondrial function and protective neuronal functions [3].

In the UK there are currently four licensed pharmacological treatments for AD. Donepezil, galanthamine and rivastigmine are acetylcholinesterase inhibitors which are prescribed to people in the mild to moderate AD, whilst the NMDA receptor antagonist memantine is licensed for moderate to severe AD. Whilst these treatments have been shown to provide benefit to symptoms and to be cost-effective [4], their benefit extends for an average of only six to 12 months, and none target the underlying AD pathology and disease processes. Whilst these treatments are enormously valuable in treating symptoms, there is an urgent need to develop better, more effective treatments designed to modify the disease process. In order to achieve this it is also imperative to better understand the precise underlying pathology in AD.

To date drug discovery and development in AD has been largely unsuccessful. The failure of several pivotal clinical trials is due to a number of factors, including the lack of breadth in molecular targets of new treatments, which have predominantly focussed on amyloid to elicit disease modification [2]. A better understanding of AD pathology and pathways is required to direct the focus of novel drugs. An additional reason for failure of candidate treatments to show benefit is thought to relate to the trial cohort characteristics, which largely include people with mild to moderate AD, whose pathology is likely to be too advanced to show benefit. Scrutiny of the literature also shows a trend to over-interpret outcomes of phase II trials showing marginal benefit, and based on theoretical mechanisms of action based on extrapolation of activity *in vitro*. This has driven the decision to take forward candidates such as tarenflurbil [5,6], dimebon [7] and semegestat [8] to larger trials which have subsequently failed. Furthermore, there is considerable heterogeneity in AD pathology, with markers ranging across inflammatory molecules and microglial activation in addition to the more specific amyloid and tau targets. This is particularly true in people over 80 where concurrent micro- and macro-vascular pathologies are also extremely common. This heterogeneity raises significant complications in cohort structure for trials to date and is likely to have contributed to the disappointing outcomes to date. A further simple, yet powerful, reason for current failures is the sheer lack of trials currently underway, which is in part due to the pharmaceutical industry’s reluctance to invest in apparently high risk AD drug development. A recent review reported only 21 trials of AD treatments are registered on the NCT or ISRCTN databases, compared to over 1,700 cancer trials [9]. In particular there are only a handful of ongoing commercially sponsored trials examining treatments for disease modification in AD, for example the ongoing RCT of the Roche immunotherapy agent Gantenerumab in people with prodromal Alzheimer’s disease [10]. Several companies are now also investigating potential new symptomatic treatments focussing on a range of targets including the noradrenergic and histaminergic systems in phase II clinical trials.

In order to improve the chances of successfully identifying and evaluating disease modifying treatments for AD it will be critical to utilise a more rigorous, evidence-based approach to drug development and clinical trials, ensuring mechanisms of action are confirmed and trial designs are optimised. This process has begun in part, with most recent trials beginning to use new diagnostic criteria which combine sensitive neuropsychological testing with biomarker changes to recruit early AD cohorts [11]. The current scarcity of trials and viable novel candidates also provides an important opportunity for drug repositioning in the field to complement more traditional industry based drug development programmes.

## 2. Drug Repositioning in Alzheimer’s Disease

Drug repositioning offers a potentially valuable and productive approach to identifying viable candidates for treatment of AD. The process, also known as drug repurposing, involves the identification of existing compounds that are already licensed for use for a different indication but which have mechanisms of action that indicate potential disease modification in AD [12]. Importantly, candidates have established safety profiles which significantly reduces the time and cost required to bring them to trial and into the clinic, although additional testing may be required in cases where effective dosage is higher for the new indication. The extent of this pre-clinical testing is outlined in Box 1, highlighting the significant advantage of repositioning where the majority of these steps have already been taken. Certain candidates may also have additional supporting evidence, for example from epidemiological studies or early clinical trials. Drug repositioning has already resulted in successes in a number of disease areas including obesity, psychosis, cancer, irritable bowel syndrome and smoking cessation [13], and is now underway in AD.

There are two main approaches to drug repositioning. The first, more straightforward approach is to investigate drugs within the mechanism of action for which they are already licensed, the most common example being the repositioning of sildenafil, previously used to treat angina, for use in erectile dysfunction [13]. The second, more innovative approach aims to identify novel targets for existing drugs, for example the repositioning of aspirin as an antithrombotic therapy following identification of its action against prothrombic thromboxane A2 activity in platelets. This approach has also been used in Parkinson’s Disease with the repurposing of amantadine, previously used to treat influenza, following discovery of its activity as an NMDA glutamate receptor antagonist [14]. Despite being more complex this second approach has the potential to identify more novel compounds. This review focuses on this broader second approach to repositioning to describe the most exciting and novel compounds. This provides a more meaningful picture of the potential for repositioning in AD, enabling the inclusion of candidates with promising pre-clinical data of novel disease-modifying actions.

A recent Delphi consensus and systematic review combined both these approaches to repositioning to identify the current most promising candidates for AD [15]. The study reviewed escalating levels of evidence for individual candidate drugs, ranging from theoretical mechanisms and *in vitro* evidence to *in vitro* studies, epidemiological findings and clinical trial data. An expert international panel followed the established Delphi consensus protocol to identify fifteen candidates with published potential for disease modification in AD. Further shortlisting included review by a panel of industry specialists and consultation with patient representatives to refine the candidates to a final priority list. Priority candidates with sufficient supporting evidence included antihypertensives, antibiotics, retinoid therapy and current treatments for diabetes (Table 1).

## 3. Treatments for Type 2 Diabetes Mellitus

The close association between AD and Type 2 diabetes mellitus (T2DM) has resulted in a number of treatments for T2DM being raised as candidate AD therapies. In addition to T2DM being a risk factor for AD, these conditions also have a common pathology in disrupted insulin signalling which is potentially a key target for therapy [16,17]. Insulin is essential in neuronal functioning and protection, and has been shown to influence tau phosphorylation [18,19,20,21]. In fact, direct administration of insulin to the brain via nasal spray results in improved cognition including attention and memory [22,23,24], and a phase II trial reported positive impacts on key AD markers including amyloid, tau and glucose metabolism [25]. These findings provide a robust rationale for investigation of agents known to regulate insulin release as AD therapies. Most promising of these are the Glucagon-Like Peptide (GLP-1) analogues currently licensed for treatment of T2DM, and which also have established brain penetration and activity in the brain [26,27,28]. *In vitro* work has demonstrated that the drugs exenatide and liraglutide influence amyloid metabolism and neuronal function through a number of different pathways including those mediated by GSK3β, caspase 3 and glutamate [29,30,31]. These effects have been confirmed in rodent models of AD where treatment with the GLP-1 analogue Val(8)GLP-1 resulted in protection of synapse activity, improved neuronal function and reduction in plaque burden [32,33]. Similar findings have been reported with liraglutide at current therapeutic dosages, and this agent also has proposed neurogenic properties [34,35].

To date no clinical evidence has been published to support the use of GLP-1 analogues in AD. However, their safety and tolerability is well established, including their use in normoglycaemic individuals, indicating their potential value outside of T2DM although they have not yet been tested in large groups of older frail people [36]. A number of phase II RCTs are ongoing and due to report in the next 12 months, emphasising the potential importance of this class of drugs in AD drug development (Table 2).

## 4. Treatments for Hypertension

There is a well-established link between hypertension in mid-life and the development of AD although the precise mechanism for this link is complex [37,38,39,40,41]. In part this is due to the impacts of overlapping and related vascular risk factors, pathology and conditions such as small vessel sub-cortical vascular disease, which are thought to play a role in AD and influence cognition [42,43]. As a result a number of antihypertensives have been highlighted as potential candidates for AD therapy. In addition to directly lowering blood pressure, these agents appear to exert independent neuroprotective effects that could result in significant pathological and symptomatic benefit.

**Table 1:** Priority candidate drugs for repositioning in AD (adapted from Corbett *et al.* 2012 [15]).

Drug class	Proposed candidate	Proposed mechanism of action	Summary of evidence
Angiotensin Receptor Blockers	Valsartan	Inhibition of inflammation, vasoconstriction and mitochondrial dysfunction Promotion of acetylcholine release Direct blockade of AT1 receptor Inhibition of Angiotensin II processing	Reduction of Aβ burden (*in vitro* / *in vitro*) Improved cognitive function (*in vitro*) Established brain penetration Epidemiological evidence for reduction of incident dementia. Two of three RCTs showed some benefit compared to placebo
Calcium Channel Blockers	Nitrendepine, nimodopine and nivaldapine	Reduction of Aβ production, burden and neurotoxicity. Differential effects indicate a novel mechanism.	Reduction of Aβ pathology and improved cell survival (*in vitro*) Cognitive improvement and reduction in pathology (*in vitro*) Clinical evidence of benefit in people with dementia, but limited in people with AD. RCTs show benefit to cognition in man in initial trials Clinical evidence to support AD risk reduction
GLP-1 analogues	Liraglutide	Neuroprotective properties involving GSK3 β and tau phosphorylation Additional effects on oxidative stress and apoptotic pathways	Reduction of intracellular APP, Aβ- and Fe^2+^-related neurodegeneration (*in vitro*) Improved synaptic plasticity and cognitive function, and reduced AD pathology (*in vitro*) Established brain penetration No epidemiological or clinical evidence.
Tetracycline antibiotics	Minocycline	Reduction of Aβ aggregation Promotion of Aβ clearance Reduction of pro-inflammatory markers	Effect on AD pathology and related inflammatory markers including microglial activation (*in vitro* / *in vitro*) Some benefit to cognitive function, although this is conflicting (*in vitro*). Benefit seen only with treatment of more than 28 days No clinical evidence although some promising findings in studies in other neurological conditions
Retinoid therapy	Acicretin	Direct effect on APP processing mediated by RXR receptor Upregulation of amyloid clearance enzymes Antioxidant regulation	Impaired retinoic acid signalling may lead to AD pathology Evidence for overall mechanistic effect (*in vitro*) Evidence for reduction in inflammation, Aβ burden and tau phosphorylation with associated cognitive benefit, although studies are conflicting (*in vitro*) No clinical data Significant safety concerns

**Table 2:** Ongoing trials in Alzheimer’s disease related to identified candidate drugs.

Drug	Phase and location	Study description	Status	Trial completion date	Clinical trial number
Acitretin	II Germany	28 days of 30 mg acitretin treatment in patients with mild to moderate Alzheimer’s Disease. The primary objective is to measure the change in APPsα levels in CSF	Recruiting	April 2011	NCT01078168
Exenatide	II USA	Exenatide in early AD or MCI, with planned follow up using sum of boxes and ADAS-COG for 36 months following treatment. MRI and CSF biomarkers as secondary measures	Recruiting	Dec 2015	NCT01255163
Liraglutide	II Denmark	26 weeks liraglutide (IV) or placebo in mild AD. Primary outcome is amyloid load by PIB PET imaging	Completed, awaiting publication	June 2013	NCT01469351
Nilvadipine	III Europe	18 month placebo controlled RCT in 500 people with AD across 18 European sites funded by the European Union	Finalizing protocol	tbc	tbc

### 4.1. Angiotensin Receptor Blockers

Angiotensin II (Ang II) acts centrally to regulate the activity of a broad range of neuronal chemicals including acetylcholine and inflammatory agents which are thought to be key to AD pathology [44,45]. *In vitro* studies have indicated that Angiotensin receptor blockers (ARBs) influence AngII via two distinct pathways, one through blockage of the AngII target, the AT_1_ receptor, and the other through augmentation of AngII processing which plays a role in cognition [46]. A number of ARBs are known to penetrate the blood brain barrier and elicit antihypertensive responses in the brain, and thus are potential AD drug candidates [47].

ARBs have performed well in *in vitro* and *in vitro* models of AD, particularly the compounds valsartan, losartan and telmisartin. Valsartan has been demonstrated to reduce Aβ accumulation and aggregation in neuronal and rodent models, with associated improvements in cognition after treatment for five months [48] although this has not been replicated in other studies [49]. Elsewhere, one *in vitro* study reported significant reduction in cerebral blood flow and plaque formation following intracranial administration of Aβ in mice pre-treated with telmisartin [50]. Interestingly, *in vitro* studies of intranasal administration of losartan resulted in a dramatic reduction of both amyloid plaque burden (3.7 fold) and inflammatory markers in mouse models of AD [51]. Overall, the evidence supports the potential of ARBs as a candidate therapy for AD, although further dose-dependence studies are required to elucidate the precise extent of the effect.

The efficacy of ARBs is also indicated by a number of epidemiological studies and RCTs. One large retrospective cohort study of 800,000 people over 65 without dementia and a further 12,000 with dementia revealed a significant reduction in dementia in people prescribed ARBs compared with other cardiovascular agenda including ACE inhibitors. Interestingly, these individuals also had a reduced rate of institutionalisation and mortality [52]. A further UK-based study reported a similar trend, with a 50% reduction in AD [53]. Although no RCTS to date have specifically focussed on the treatment of AD with ARBs a number of RCTs in people with cardiovascular disease and diabetes have included cognitive outcome measures, and indicate benefit. These include the ONTARGET and TRANSCEND studies in 16,000 and 5,000 people, respectively, which evaluated the benefit of telmisartan, and the SCOPE study of 4937 people which investigated candesartan. All included the Mini Mental State Examination (MMSE) as an outcome measure. The ONTARGET study reported a reduction in decline in MMSE score but TRANSCEND and SCOPE reported no difference despite showing benefit in cardiovascular outcomes [54,55]. However, analysis of outcomes for 2,020 people in the SCOPE cohort with lower MMSE baseline scores did reveal a slower decline in the treatment group compared to placebo [56]. These findings from studies in man are conflicting and difficult to interpret. However, the indications from their outcomes are sufficient to warrant a large RCT to specifically provide evidence regarding the benefit of ARBs in AD. Prior to this definitive trial, further *in vitro* work would be required to identify the most suitable ARB and dosage.

### 4.2. Calcium Channel Blockers

Calcium Channel Blockers are a commonly used antihypertensive which elicit a vasodilatory effect on vascular tissue, and this effect has been recorded in the brain [57,58,59]. There is considerable clinical data to support the use of CCBs in AD, which has provided the rationale for further *in vitro* and *in vitro* studies to elucidate the mechanism. This work has reported the reduction of Aβ production, aggregation and neurotoxicity and improved neuronal function in the presence of CCBs both in vtiro and *in vitro* [60,61,62,63]. Amlodipine and nivaldipine have shown particular promise in this work, including in transgenic AD rodents, showing improvement in cognitive measures including learning and memory although effective dosages were usually far higher than those licensed for therapeutic use [64]. Nivaldipine out-performed amlodipine in additional *in vitro* studies where approximate therapeutic-level doses were used [65]. Ispradipine has also shown promise in initial *in vitro* and *in vitro* work [62,66]. This evidence base indicates nivaldipine as the most promising CCB candidate for AD therapy, and also appears to show that the underlying mechanism is independent of the anti-hypertensive action due to the differential effects seen with the different CCB agents.

Clinical evidence of the efficacy of CCBs in dementia and AD include a number of RCTs in people with dementia. Although the majority of these RCTs are small, of 12 weeks or less and did not include analysis of impact on disease pathology, a Cochrane review which analysed the findings of 15 RCTs of nimodipine reported significant impact on cognition [67]. Two trials have specifically investigated the effect in AD, the largest including over 1,000 people and reporting significant improvement in cognition and overall clinical status at 12 weeks, with sustained cognitive benefit at 24 weeks [68]. The only study of nivaldipine to date was a small initial RCT which reported good tolerability of the agent in people with AD [69]. Interestingly, findings indicate that non-ApoE4 carriers respond better to treatment, and this is now under further investigation in a large-scale RCT [70]. Clinical evidence from epidemiological studies, including a cohort of 3,000 people over 74, also appears to support the efficacy of dihydropyridine CCBs in reducing or delaying the development of AD [71]. This is supported by the recent SYST-EUR RCT which reported a 55% reduction in incident dementia in people treated with nitrendipine over long-term follow-up [72].

There is therefore promising evidence to support the use of CCBs in both treating and preventing AD, although the underlying mechanism is not yet clear. Taken together, nitrendipine, nimodipine and nilvadipine appear to be the best candidates for future investigation.

## 5. Antibiotics

The tetracycline antibiotic minocycline has been proposed as a candidate for AD therapy due to its blood-brain barrier penetrative properties and a promising evidence base in clinical studies outside of AD. This class of compounds also has a good safety profile in older people and elicits few drug-drug interactions. The potential value of minocycline is supported by *in vitro* work which has demonstrated reduction in amyloid pathology. *In vitro* studies have reported decreased inflammatory markers and activation of microglia [73,74,75], and improvement in behavioural symptoms in transgenic mouse models of AD and T2DM [76,77,78]. Importantly however, a number of studies have not observed any benefit in pathology with minocycline treatment of less than 28 days, indicating a probable threshold for effective disease modification [75,79,80]. Furthermore, to date no pre-clinical studies of minocycline have administered a dosage that is within the current equivalent licensed range. However, toxicology information is already available at these higher dose ranges, and given the established good tolerability profile of the drug, this is unlikely to present a major obstacle to its use.

Minocycline has been evaluated in clinical trials in a range of neurodegenerative conditions with conflicting results. RCTs of treatment for amytrophic lateral sclerosis and Huntington’s disease reported no benefit [81,82,83,84], whilst preliminary studies in Parkinson’s disease have provided sufficiently promising data to progress to phase III trial [85]. It is likely that these differential outcomes reflect the more focussed neuroprotective activity elicited by minocycline rather than a broader effect that would result in benefit to a wider range of neurodegenerative conditions.

The antifungal medication clioquinol was proposed as a candidate for repositioning in AD following *in vitro* and *in vitro* evidence of its ability to promote amyloid clearance through its activity as a metal chelator [86]. However, the drug performed poorly in initial clinical trials and a phase II trial was halted due to concerns about toxicity. A derivative of this drug, PBT2, has now been developed and taken to trial where it has shown an improved safety profile but to date has failed to confer a significant improvement in cognition [87].

## 6. Retinoid Therapy

Retinoid therapy involves treatment with compounds to promote activity of retinoic acid receptors, a process that is integral to neuronal function and repair. These drugs are frequently used to treat skin conditions but evidence from mechanistic studies indicates they may be a promising candidate for repositioning due to suggested impaired retinoic acid signalling in AD [88,89,90]. Furthermore, retinoids are known to promote activity of pathways involved in amyloid processing, neurogenesis and neuronal function including acting as antioxidants and inflammatory inhibitors [91,92,93,94]. However, there is only limited *in vitro* and *in vitro* evidence to demonstrate how these mechanisms might impact on AD.

One *in vitro* study has demonstrated the reversal of the impaired retinoic acid signalling seen in AD following administration of all-trans retinoic acid. Similarly positive results have been reported *in vitro*, including benefit to behaviour, memory and learning. However, this work was based on treatment with all-trans retinoic acid at dosages that cause an unacceptably high number of adverse effects [95]. Subsequent *in vitro* work has reported that treatment with the synthetic retinoid acitretin confers similar benefit to pathology and cognition in rodents [96], with a further study demonstrating disease-modification following administration of tamibarotene, although the effect on behaviour was not measured [97]. The RXR agonist bexarotene, currently licensed for treatment of cutaneous T-cell lymphoma, has demonstrated significant reductions in amyloid processing, but not pathology, and related cognitive improvements in mouse models of AD [98]. However, again the dose used was three times higher than is currently used in man. Given the significant safety concerns associated with this therapy, this would likely be an obstacle to drug development. Although there is a plausible rationale for retinoid therapy as an AD treatment, it will be critical to elucidate the precise mode of action prior in further pre-clinical work prior to taking it forward to clinical trial. A critical additional consideration is the considerable adverse effect profile of this class of drugs. Despite the reduced relevance of certain effects, such as teratogenesis, due to the average age of the target patient group, side effects include severe thirst, mucosal drying, headache and abnormal lipids which could severely impact tolerability. Interestingly, a recent review consulted focus groups of people with dementia and carers regarding their perceived acceptable threshold for side effects for an effective AD treatment, and revealed a surprisingly high tolerance for adverse events.

## 7. Taking Forward the Opportunity for Drug Repositioning

Drug repositioning offers an exciting and potentially impactful route to drug development in AD. The landscape of increasing openness to this approach has led to more opportunities for large-scale funding to utilise high- and medium-throughput laboratory approaches to identify large numbers of potential drug candidates through combinations of transcriptomics and microarray techniques alongside established *in vitro* and *in vitro* models. Global gene expression offers a high content quantitative methodology to compare biological states. This underlies the Broad institute’s connectivity map (CMAP) project [99], which established a database of the transcriptional profiles associated with a spectrum of drugs and drug-like compounds. One salient application of CMAP is the marrying of disease state to drug through an anti-correlation of the respective transcriptional profiles [100]. Recently, a searchable platform-independent expression database (SPIED) has extended the CMAP methodology to cover transcriptional data in the public domain [101]. Interestingly, SPIED has revealed conserved patterns of gene expression associated with neurodegenerative disease. In particular, AD associated gene expression changes were shown to be consistent across multiple independent studies and a core set of highly regulated genes showed a conspicuous anti-correlation with a set of drugs with established neuroprotective activity [101]. Among the predicted AD therapeutics was galantamine, currently prescribed for early stage AD. Other significant anti-correlating drugs were the flavones apigenin and luteolin, which have been reported to be neuroprotective [102,103]. Two kinase inhibitors H7 and GW8510, the alkaloid harmine, the dopamine reuptake inhibitor nomifensine and the acetylcholine receptor agonist carbachol have also been identified. Another intriguing “hit” was metacycline a tetracycline antibiotic, the same class of antibiotic implicated in AD therapy as mentioned above. These remarkable observations highlight the potential of disease associated transcriptomes as a basis for drug repositioning in AD. To further support this work, newly available large drug libraries provide comprehensive safety data provide a valuable source of compounds. This raises the possibility of driving forward a greater number of compounds entering initial clinical studies, and therefore the potential of a novel therapy, much more quickly than would have been possible a decade ago.

A number of promising candidate compounds emerging from this body of work have now entered preliminary or large scale clinical trials in AD. These include a phase III trial of nivaldipine currently commencing in the European Union, and phase II trials of the ARB losartan, minocycline, acicretin and two GLP-1 analogues liraglutide and exendin currently underway in the US and Europe.

[Box 1] Recurrent lines of investigation for preclinical and early clinical investigation required to advance drugs to phase III clinical trials.

- Determination of dose-response relationships in animal models of AD.
- Highest dose that can be safely administered on the basis of current pre-clinical and clinical data
- Understanding the effect and safety associated with chronic administration of drug.
- Understanding pharmacokinetics and pharmacodynamics in animal models and their relationship to man.
- Understanding in the course of disease progression when the optimal time to commence treatment may be to gain maximum efficacy.
- For drug classes where more than one agent in class is available; detailed intra-class comparability data is required.
- Measurement of suitable biomarker changes in phase II clinical trials.
  ○ Changes in CSF biomarkers (AP1-40, AP1-41, phosphor tau, inflammatory markers
  ○ Changes in amyloid load using 13C –PCB PET imaging
  ○ Changes in microglial activation and brain glucose metabolism using PET imaging
  ○ Changes in hippocampal atrophy using serial MRI
  ○ Changes in inflammatory markers in blood and CSF

## 8. Conclusions

Drug repositioning offers an exciting opportunity to develop novel, effective treatments for AD in a fraction of the time and at far lower cost than required for traditional drug discovery routes. New emerging technologies will soon enable high-throughput screening and compound identification based on transcriptomics and biomarker profiles, enabling a more targeted approach. With several repositioning candidates for AD already in phase II and III trials, and ongoing improvements in clinical trial design, the movement towards drug repositioning is gaining momentum and building the required infrastructure for successful drug development in this important field.

## References

[1] Wimo A., Prince M. World Alzheimer Report 2010—The Global Economic Impact of Dementia. http://www.eldis.org/go/topics&id=56368&type=Document#.UlTiyqxX8n1.

[2] Karran E., Mercken M., De Strooper B. (2011). The amyloid cascade hypothesis for Alzheimer’s disease: An appraisal for the development of therapeutics. Nat. Rev. Drug Discov..

[3] Ballard C., Gauthier S., Corbett A., Brayne C., Aarsland D., Jones E. (2011). Alzheimer’s disease. Lancet.

[4] Ballard C., Corbett A., Sharp S. (2011). Aligning the evidence with practice: NICE guidelines for drug treatment of Alzheimer’s disease. Expert. Rev. Neurother..

[5] Wilcock G.K., Black S.E., Hendrix S.B., Zavitz K.H., Swabb E.A., Laughlin M.A. (2008). Tarenflurbil Phase II Study investigators. Efficacy and safety of tarenflurbil in mild to moderate Alzheimer’s disease: A randomised phase II trial. Lancet Neurol..

[6] Green R.C., Schneider L.S., Amato D.A., Beelen A.P., Wilcock G., Swabb E.A., Zavitz K.H., Tarenflurbil Phase 3 Study Group (2009). Effect of tarenflurbil on cognitive decline and activities of daily living in patients with mild Alzheimer disease: A randomized controlled trial. JAMA.

[7] Zhang S., Hedskog L., Petersen C.A., Winblad B., Ankarcrona M. (2010). Dimebon (latrepirdine) enhances mitochondrial function and protects neuronal cells from death. J. Alzheimers. Dis..

[8] D’Onofrio G., Panza F., Frisardi V., Solfrizzi V., Imbimbo B.P., Paroni G., Cascavilla L., Seripa D., Pilotto A. (2012). Advances in the identification of gamma-secretase inhibitors for the treatment of Alzheimer’s disease. Expert. Opin. Drug Disco..

[9] Mangialasche F., Solomon A., Winblad B., Mecocci P., Kivipelto M. (2009). Alzheimer’s disease: Clinical trials and drug development. Lancet Neurol..

[10] Hoffmann-La Roche A Study of Gantenerumab in Patients With Prodromal Alzheimer’s Disease. http://clinicaltrials.gov/ct2/show/NCT01224106.

[11] Dubois B., Feldman H.H., Jacova C., Dekosky S.T., Barberger-Gateau P., Cummings J., Delacourte A., Galasko D., Gauthier S., Jicha G. (2007). Research criteria for the diagnosis of Alzheimer's disease: Revising the NINCDS-ADRDA criteria. Lancet Neurol..

[12] Sirota M., Dudley J.T., Kim J., Chiang A.P., Morgan A.A., Sweet-Cordero A., Sage J., Butte A.J. (2011). Discovery and Preclinical Validation of Drug Indications Using Compendia of Public Gene Expression Data. Sci. Transl. Med..

[13] Ashburn T.T., Thor K.B. (2004). Drug repositioning: Identifying and developing new uses for existing drugs. Nat. Rev. Drug Discov..

[14] Hubsher G., Haider M., Okun M.S. (2012). Amantadine: The journey from fighting flu to treating Parkinson disease. Neurology.

[15] Corbett A., Pickett J., Burns A., Corcoran J., Dunnett S.B., Edison P., Hagan J.J., Holmes C., Jones E., Katona C. (2012). Drug repositioning for Alzheimer’s disease. Nat. Rev. Drug Discov..

[16] Schrijvers E.M., Witteman J.C., Sijbrands E.J., Hofman A., Koudstaal P.J., Breteler M.M. (2010). Insulin metabolism and the risk of Alzheimer disease: The Rotterdam Study. Neurology.

[17] Moloney A.M., Griffin R.J., Timmons S., O’Connor R., Ravid R., O’Neill C. (2010). Defects in IGF-1 receptor, insulin receptor and IRS-1/2 in Alzheimer’s disease indicate possible resistance to IGF-1 and insulin signalling. Neurobiol. Aging.

[18] Hoyer S. (2009). Glucose metabolism and insulin receptor signal transduction in Alzheimer disease. Eur J. Pharmacol..

[19] Li L., Hölscher C. (2007). Common pathological processes in Alzheimer Disease and Type 2 Diabetes: A review. Brain Res. Rev..

[20] Li Z.G., Zhang W., Sima A.A. (2007). Alzheimer-like changes in rat models of spontaneous diabetes. Diabetes.

[21] Carro E., Torres A.I. (2004). The role of insulin and insulin-like growth factor I in the molecular and cellular mechanisms underlying the pathology of Alzheimer’s disease. Eur. J. Pharmacol..

[22] Reger M.A., Watson G.S., Green P.S., Baker L.D., Cholerton B., Fishel M.A., Plymate S.R., Cherrier M.M., Schellenberg G.D., Frey W.H., Craft S. (2008). Intranasal insulin administration dose-dependently modulates verbal memory and plasma amyloid-beta in memory-impaired older adults. J. Alzheimers Dis..

[23] Reger M.A., Watson G.S., Green P.S., Wilkinson C.W., Baker L.D., Cholerton B., Fishel M.A., Plymate S.R., Breitner J.C., DeGroodt W. (2008). Intranasal insulin improves cognition and modulates beta-amyloid in early AD. Neurology.

[24] Baker L.D., Cross D.J., Minoshima S., Belongia D., Watson G.S., Craft S. (2011). Insulin resistance and Alzheimer-like reductions in regional cerebral glucose metabolism for cognitively normal adults with prediabetes or early type 2 diabetes. Arch. Neurol..

[25] Craft S., Baker L.D., Montine T.J., Minoshima S., Watson G.S., Claxton A., Arbuckle M., Callaghan M., Tsai E., Plymate S.R. (2012). Intranasal insulin therapy for Alzheimer disease and amnestic mild cognitive impairment: A pilot clinical trial. Arch. Neurol..

[26] Kastin A.J., Akerstrom V., Pan W. (2002). Interactions of glucagon-like peptide-1 (GLP-1) with the blood-brain barrier. J. Mol. Neurosci..

[27] Kastin A.J., Akerstrom V. (2003). Entry of exendin-4 into brain is rapid but may be limited at high doses. Int. J. Obes. Relat. Metab. Disord..

[28] McClean P., Pathasarthy V., Gault V., Holscher C. Liraglutide, a novel GLP-1 analogue, prevents the impairment of learning and LTP in an APP/PS-1 mouse model of Alzheimer’s disease. Presented at the Society for Neuroscience (SfN).

[29] Perry T., Lahiri D.K., Sambamurti K., Chen D., Mattson M.P., Egan J.M., Greig N.H. (2003). Glucagon-like peptide-1 decreases endogenous amyloid-beta peptide (A beta) levels and protects hippocampal neurons from death induced by A beta and iron. J. Neurosci. Res..

[30] Li L., Zhang Z.F., Holscher C., Gao C., Jiang Y.H., Liu Y.Z. (2012). (Val^8^) glucagon-like peptide-1 prevents tau hyperphosphorylation, impairment of spatial learning and ultra-structural cellular damage induced by streptozotocin in rat brains. Euro. J. Pharmacol..

[31] Wang X.H., Li L., Hölscher C., Pan Y.F., Chen X.R., Qi J.S. (2010). Val8-glucagon-like peptide-1 protects against Aβ1–40-induced impairment of hippocampal late-phase long-term potentiation and spatial learning in rats. Neuroscience.

[32] Radde R., Bolmont T., Kaeser S.A., Coomaraswamy J., Lindau D., Stoltze L., Calhoun M.E., Jäggi F., Wolburg H., Gengler S. (2006). Abeta42-driven cerebral amyloidosis in transgenic mice reveals early and robust pathology. EMBO Rep..

[33] Gengler S., McClean P.L., McCurtin R., Gault V.A., Hölscher C. (2012). Val(8)GLP-1 rescues synaptic plasticity and reduces dense core plaques in APP/PS1 mice. Neurobiol. Aging.

[34] McClean P.L., Parthsarathy V., Faivre E., Hölscher C. (2011). The Diabetes Drug Liraglutide Prevents Degenerative Processes in a Mouse Model of Alzheimer’s Disease. J. Neurosci..

[35] Hamilton A., Patterson S., Porter D., Gault V.A., Hölscher C. (2011). Novel GLP-1 mimetics developed to treat type 2 diabetes promote progenitor cell proliferation in the brain. J. Neurosci. Res..

[36] Astrup A., Rössner S., van Gaal L., Rissanen A., Niskanen L., Al-Hakim M., Madsen J., Rasmussen M.F., Lean M.E.J. (2009). Effects of liraglutide in the treatment of obesity: A randomised, double-blind, placebo-controlled study. Lancet.

[37] Yoshitake T., Kiyohara Y., Kato I., Ohmura T., Iwamoto H., Nakayama K., Ohmori S., Nomiyama K., Kawano H., Ueda K. (1995). Incidence and risk factors of vascular dementia and Alzheimer’s disease in a defined elderly Japanese population: the Hisayama Study. Neurology.

[38] Skoog I., Lernfelt B., Landahl S., Palmertz B., Andreasson L.A., Nilsson L., Persson G., Odén A., Svanborg A. (1996). 15-year longitudinal study of blood pressure and dementia. Lancet.

[39] Launer L.J., Ross G.W., Petrovitch H., Masaki K., Foley D., White L.R., Havlik R.J. (2000). Midlife blood pressure and dementia: The Honolulu-Asia aging study. Neurobiol. Aging.

[40] Posner H.B., Tang M.X., Luchsinger J., Lantigua R., Stern Y., Mayeux R. (2002). The relationship of hypertension in the elderly to AD, vascular dementia, and cognitive function. Neurology.

[41] Qiu C., Winblad B., Fratiglioni L. (2005). The age-dependent relation of blood pressure to cognitive function and dementia. Lancet Neurol..

[42] Dickstein D.L., Walsh J., Brautigam H., Stockton S.D., Gandy S., Hof P.R. (2010). Role of Vascular Risk Factors and Vascular Dysfunction in Alzheimer’s Disease. Mt. Sinai J. Med..

[43] Snowdon D.A., Greiner L.H., Mortimer J.A., Riley K.P., Greiner P.A., Markesbery W.R. (1997). Brain infarction and the clinical expression of Alzheimer disease—The nun study. JAMA.

[44] Kehoe P.G., Passmore P.A. (2012). The renin-angiotensin system and antihypertensive drugs in Alzheimer’s disease: Current standing of the angiotensin hypothesis?. J. Alzheimers Dis..

[45] Kehoe P.G., Miners S., Love S. (2009). Angiotensins in Alzheimer’s disease—Friend or foe?. Trends Neurosci..

[46] Wright J.W., Harding J.W. (2011). Brain renin-angiotensin-A new look at an old system. Prog. Neurobiol..

[47] Culman J., Blume A., Gohlke P., Unger T. (2002). The renin-angiotensin system in the brain: Possible therapeutic implications for AT(1)-receptor blockers. J. Hum. Hypertens..

[48] Wang J., Ho L., Chen L., Zhao Z., Zhao W., Qian X., Humala N., Seror I., Bartholomew S., Rosendorff C., Pasinetti G.M. (2007). Valsartan lowers brain beta-amyloid protein levels and improves spatial learning in a mouse model of Alzheimer disease. J. Clin. Invest..

[49] Ferrington L., Miners J.S., Palmer L.E., Bond S.M., Povey J.E., Kelly P.A., Love S., Horsburgh K.J., Kehoe P.G. (2011). Angiotensin II-inhibiting drugs have no effect on intraneuronal Abeta or oligomeric Abeta levels in a triple transgenic mouse model of Alzheimer’s disease. Am. Transl. Res..

[50] Mogi M., Li J.M., Tsukuda K., Iwanami J., Min L.J., Sakata A., Fujita T., Iwai M., Horiuchi M. (2008). Telmisartan prevented cognitive decline partly due to PPAR-gamma activation. Biochem. Biophys. Res. Commun..

[51] Danielyan L., Klein R., Hanson L.R., Buadze M., Schwab M., Gleiter C.H., Frey W.H. (2010). Protective Effects of Intranasal Losartan in the APP/PS1 Transgenic Mouse Model of Alzheimer Disease. Rejuvenation Res..

[52] Li N.C., Lee A., Whitmer R.A., Kivipelto M., Lawler E., Kazis L.E., Wolozin B. (2010). Use of angiotensin receptor blockers and risk of dementia in a predominantly male population: Prospective cohort analysis. BMJ.

[53] Davies N., Kehoe P., Ben-Shlomo Y., Martin R. (2011). Associations Of Angiotensin-Ii Receptor Blockers And Ace Inhibitors With Alzheimer’s Disease: A Nested Case-Control Study Within The Uk General Practice Research Database. J. Epidemiol. Community Health.

[54] Anderson C., Teo K., Gao P., Arima H., Dans A., Unger T., Commerford P., Dyal L., Schumacher H., Pogue J. (2011). Renin-angiotensin system blockade and cognitive function in patients at high risk of cardiovascular disease: Analysis of data from the ONTARGET and TRANSCEND studies. Lancet Neurol..

[55] Lithell H., Hansson L., Skoog I., Elmfeldt D., Hofman A., Olofsson B., Trenkwalder P., Zanchetti A. (2003). The Study on cognition and prognosis in the elderly (SCOPE): Principal results of a randomized double-blind intervention trial. J. Hypertens..

[56] Skoog I., Lithell H., Hansson L., Elmfeldt D., Hofman A., Olofsson B., Trenkwalder P., Zanchetti A. (2005). Effect of baseline cognitive function and anti hypertensive treatment on cognitive and cardiovascular outcomes: Study on COgnition and Prognosis in the Elderly (SCOPE). Am. J. Hypertens..

[57] Landmark K., Forsman M., Lindberg K., Ryman T., Martmann-Moe K., Haaverstad S., Wiel S. (1995). Nitrendipine And Mefruside In Elderly Hypertensives—Effects On Blood-Pressure, Cardiac-Output, Cerebral Blood-Flow And Metabolic Parameters. J. Hum. Hypertens..

[58] Hanyu H., Hirao K., Shimizu S., Iwamoto T., Koizumi K., Abe K. (2007). Favourable effects of nilvadipine on cognitive function and regional cerebral blood flow on SPECT in hypertensive patients with mild cognitive impairment. Nucl. Med. Commun..

[59] Forsman M., Olsnes B.T., Semb G., Steen P.A. (1990). Effects Of Nimodipine On Cerebral Blood-Flow And Neuropsychological Outcome After Cardiac-Surgery. Br. J. Anaesth..

[60] Zhao W., Wang J., Ho L., Ono K., Teplow D.B., Pasinetti G.M. (2009). Identification of Antihypertensive Drugs Which Inhibit Amyloid-beta Protein Oligomerization. J. Alzheimers Dis..

[61] Bachmeier C., Beaulieu-Abdelahad D., Mullan M., Paris D. (2011). Selective dihydropyiridine compounds facilitate the clearance of beta-amyloid across the blood-brain barrier. Eur. J. Pharmacol..

[62] Anekonda T.S., Quinn J.F., Harris C., Frahler K., Wadsworth T.L., Woltjer R.L. (2011). L-type voltage-gated calcium channel blockade with isradipine as a therapeutic strategy for Alzheimer’s disease. Neurobiol. Dis..

[63] Li N., Liu B., Dluzen D.E., Jin Y. (2007). Protective effects of ginsenoside Rg(2) against glutamate-induced neurotoxicity in PC12 cells. J. Ethnopharmacol..

[64] Paris D., Bachmeier C., Patel N., Quadros A., Volmar C.H., Laporte V., Ganey J., Beaulieu-Abdelahad D., Ait-Ghezala G., Crawford F., Mullan M.J. (2011). Selective Antihypertensive Dihydropyridines Lower A beta Accumulation by Targeting both the Production and the Clearance of A beta across the Blood-Brain Barrier. Mol. Med..

[65] Iwasaki K., Egashira N., Takagaki Y., Yoshimitsu Y., Hatip-Al-Khatib I., Mishima K., Fujiwara M. (2007). Nilvadipine prevents the impairment of spatial memory induced by cerebral ischemia combined with beta-amyloid in rats. Bio. Pharm. Bull..

[66] Copenhaver P.F., Anekonda T.S., Musashe D., Robinson K.M., Ramaker J.M., Swanson T.L., Wadsworth T.L., Kretzschmar D., Woltjer R.L., Quinn J.F. (2011). A translational continuum of model systems for evaluating treatment strategies in Alzheimer’s disease: Isradipine as a candidate drug. Dis. Model. Mech..

[67] Lopez-Arrieta J.M., Birks J. (2002). Nimodipine for primary degenerative, mixed and vascular dementia. Cochrane Database Syst. Rev..

[68] Morich F.J., Bieber F., Lewis J.M., Kaiser L., Cutler N.R., Escobar J.I., Willmer J., Petersen R.C., Reisberg B. (1996). Nimodipine in the treatment of probable Alzheimer’s disease. Results of two multicentre trials. Clin. Drug Invest..

[69] Kennelly S.P., Abdullah L., Paris D., Parish J., Mathura V., Mullan M., Crawford F., Lawlor B.A., Kenny R.A. (2011). Demonstration of safety in Alzheimer’s patients for intervention with an anti-hypertensive drug Nilvadipine: Results from a 6-week open label study. Int. J. Geriatr. Psychiatry.

[70] Kennelly S., Abdullah L., Kenny R.A., Mathura V., Luis C.A., Mouzon B., Crawford F., Mullan M., Lawlor B. (2012). Apolipoprotein E genotype-specific short-term cognitive benefits of treatment with the antihypertensive nilvadipine in Alzheimer’s patients—An open-label trial. Int. J. Geriatr. Psychiatry.

[71] Khachaturian A.S., Zandi P.P., Lyketsos C.G., Hayden K.M., Skoog I., Norton M.C., Tschanz J.T., Mayer L.S., Welsh-Bohmer K.A., Breitner J.C. (2006). Antihypertensive medication use and incident Alzheimer disease—The Cache County Study. Arch. Neurol..

[72] Forette F., Seux M.L., Staessen J.A., Thijs L., Babarskiene M.R., Babeanu S., Bossini A., Fagard R., Gil-Extremera B., Laks T. (2002). The prevention of dementia with antihypertensive treatment: New evidence from the Systolic Hypertension in Europe (Syst-Eur) study. Arch. Int. Med..

[73] Forloni G., Colombo L., Girola L., Tagliavini F., Salmona M. (2001). Anti-amyloidogenic activity of tetracyclines: Studies *in vitro*. FEBS Lett..

[74] Ryu J.K., Franciosi S., Sattayaprasert P., Kim S.U., McLarnon J.G. (2004). Minocycline inhibits neuronal death and glial activation induced by beta-amyloid peptide in rat hippocampus. Glia.

[75] Fan R., Xu F., Previti M.L., Davis J., Grande A.M., Robinson J.K., van Nostrand W.E. (2007). Minocycline reduces microglial activation and improves behavioral deficits in a transgenic model of cerebral microvascular amyloid. J. Neurosci..

[76] Parachikova A., Vasilevko V., Cribbs D.H., LaFerla F.M., Green K.N. (2010). Reductions in Amyloid-beta-Derived Neuroinflammation, with Minocycline, Restore Cognition but do not Significantly Affect Tau Hyperphosphorylation. J. Alzheimers. Dis..

[77] Cuello A.C., Ferretti M.T., Leon W.C., Iulita M.F., Melis T., Ducatenzeiler A., Bruno M.A., Canneva F. (2010). Early-Stage Inflammation and Experimental Therapy in Transgenic Models of the Alzheimer-Like Amyloid Pathology. Neurodegener. Dis..

[78] Cai Z.Y., Zhao Y., Yao S.T., Zhao B. (2011). Increases in beta-amyloid protein in the hippocampus caused by diabetic metabolic disorder are blocked by minocycline through inhibition of NF-kappa B pathway activation. Pharmacol. Rep..

[79] Garcia-Alloza M., Prada C., Lattarulo C., Fine S., Borrelli L.A., Betensky R., Greenberg S.M., Frosch M.P., Bacskai B.J. (2009). Matrix metalloproteinase inhibition reduces oxidative stress associated with cerebral amyloid angiopathy *in vitro* in transgenic mice. J. Neurochem..

[80] Malm T.M., Magga J., Kuh G.F., Vatanen T., Koistinaho M., Koistinaho J. (2008). Minocycline Reduces Engraftment and Activation of Bone Marrow-Derived Cells but Sustains Their Phagocytic Activity in a Mouse Model of Alzheimer’s Disease. Glia.

[81] Gordon P.H., Moore D.H., Miller R.G., Florence J.M., Verheijde J.L., Doorish C., Hilton J.F., Spitalny G.M., MacArthur R.B., Mitsumoto H. (2007). Efficacy of minocycline in patients with amyotrophic lateral sclerosis: A phase III randomised trial. Lancet. Neurol..

[82] Bonelli R.M., Heuberger C., Reisecker F. (2003). Minocycline for Huntington’s disease: An open label study. Neurology.

[83] Bonelli R.M., Hodl A.K., Hofmann P., Kapfhammer H.P. (2004). Neuroprotection in Huntingtons disease: A 2-year study on minocycline. Int. Clin. Psychopharmacol..

[84] Thomas M., Ashizawa T., Jankovic J. (2004). Minocycline in Huntington’s disease: A pilot study. Mov. Disord..

[85] The NINDS NET-PD Investigators (2008). A pilot clinical trial of creatine and minocycline in early Parkinson disease: 18-Month results. Clin. Neuropharmacol..

[86] Zhang Y.H., Raymick J., Sarkar S., Lahiri D.K., Ray B., Holtzman D., Dumas M., Schmued L.C. (2013). Efficacy and toxicity ofclioquinoltreatment and A-β42 inoculation in the APP/PSI mouse model of Alzheimer’s disease. Curr. Alzheimer Res..

[87] Sampson E.L., Jenagaratnam L., McShane R. (2012). Metal protein attenuating compounds for the treatment of Alzheimer’s dementia. Cochrane Database Syst Rev..

[88] Goodman A.B., Pardee A.B. (2003). Evidence for defective retinoid transport and function in late onset Alzheimer’s disease. Proc. Natl. Acad. Sci. USA.

[89] Corcoran J.P.T., So P.L., Maden M. (2004). Disruption of the retinoid signalling pathway causes a deposition of amyloid beta in the adult rat brain. Eur. J. Neurosci..

[90] Husson M., Enderlin V., Delacourte A., Ghenimi N., Alfos S., Pallet V., Higueret P. (2006). Retinoic acid normalizes nuclear receptor mediated hypo-expression of proteins involved in β-amyloid deposits in the cerebral cortex of vitamin A deprived rats. Neurobiol. Dis..

[91] Melino G., Draoui M., Bernardini S., Bellincampi L., Reichert U., Cohen P. (1996). Regulation by retinoic acid of insulin-degrading enzyme and of a related endoprotease in human neuroblastoma cell lines. Cell. Growth Differ..

[92] So P.L., Yip P.K., Bunting S., Wong L.F., Mazarakis N.D., Hall S., McMahon S., Maden M., Corcoran J.P. (2006). Interactions between retinoic acid, nerve growth factor and sonic hedgehog signalling pathways in neurite outgrowth. Dev. Biol..

[93] Lee H.P., Casadesus G., Zhu X., Lee H.G., Perry G., Smith M.A., Gustaw-Rothenberg K., Lerner A. (2009). All-trans retinoic acid as a novel therapeutic strategy for Alzheimer’s disease. Expert Rev. Neurother..

[94] Shudo K., Fukasawa H., Nakagomi M., Yamagata N. (2009). Towards Retinoid Therapy for Alzheimer’s Disease. Curr. Alzheimer Res..

[95] Ding Y., Qiao A., Wang Z., Goodwin J.S., Lee E.S., Block M.L., Allsbrook M., McDonald M.P., Fan G.H. (2008). Retinoic Acid Attenuates beta-Amyloid Deposition and Rescues Memory Deficits in an Alzheimer’s Disease Transgenic Mouse Model. J. Neurosci..

[96] Tippmann F., Hundt J., Schneider A., Endres K., Fahrenholz F. (2009). Up-regulation of the α-secretase ADAM10 by retinoic acid receptors and acitretin. FASEB J..

[97] Kawahara K., Nishi K., Suenobu M., Ohtsuka H., Maeda A., Nagatomo K., Kuniyasu A., Staufenbiel M., Nakagomi M., Shudo K., Nakayama H. (2009). Oral Administration of Synthetic Retinoid Am80 (Tamibarotene) Decreases Brain beta-Amyloid Peptides in APP23 Mice. Biol. Pharm. Bull..

[98] Cramer P.E., Cirrito J.R., Wesson D.W., Lee C.Y., Karlo J.C., Zinn A.E., Casali B.T., Restivo J.L., Goebel W.D., James M.J. (2012). ApoE-directed therapeutics rapidly clear β-amyloid and reverse deficits in AD mouse models. Science.

[99] Lamb J., Crawford E.D., Peck D., Modell J.W., Blat I.C., Wrobel M.J., Lerner J., Brunet J.P., Subramanian A., Ross K.N. (2006). The Connectivity Map: Using gene-expression signatures to connect small molecules, genes, and disease. Science.

[100] Wei G., Twomey D., Lamb J., Schlis K., Agarwal J., Stam R.W., Opferman J.T., Sallan S.E., den Boer M.L., Pieters R. (2006). Gene expression-based chemical genomics identifies rapamycin as a modulator of MCL1 and glucocorticoid resistance. Cancer Cells.

[101] Williams G. (2012). A searchable cross-platform gene expression database reveals connections between drug treatments and disease. BMC Genomics.

[102] Guo D.J., Li F., Yu P.H., Chan S.W. (2013). Neuroprotective effects of luteolin against apoptosis induced by 6-hydroxydopamine on rat pheochromocytoma PC12 cells. Pharm. Biol..

[103] Taupin P. (2009). Apigenin and related compounds stimulate adult neurogenesis. Mars, Inc., the Salk Institute for Biological Studies: WO2008147483. Expert Opin. Ther. Pat..

